# Native mass spectrometry goes more native: investigation of membrane protein complexes directly from SMALPs†
†Electronic supplementary information (ESI) available: Experimental details, additional information on methods. See DOI: 10.1039/c8cc06284f


**DOI:** 10.1039/c8cc06284f

**Published:** 2018-11-19

**Authors:** Nils Hellwig, Oliver Peetz, Zainab Ahdash, Igor Tascón, Paula J. Booth, Vedrana Mikusevic, Marina Diskowski, Argyris Politis, Yvonne Hellmich, Inga Hänelt, Eamonn Reading, Nina Morgner

**Affiliations:** a Institute of Physical and Theoretical Chemistry , Goethe University Frankfurt , Max-von-Laue-Straße 7 , 60438 Frankfurt , Germany . Email: morgner@chemie.uni-frankfurt.de; b Department of Chemistry , King's College London , 7 Trinity Street , SE1 1DB , London , UK; c Institute of Biochemistry , Goethe University Frankfurt , Max-von-Laue-Straße 9 , 60438 Frankfurt , Germany

## Abstract

Other than more widely used methods, the use of styrene maleic acid allows the direct extraction of membrane proteins from the lipid bilayer into SMALPs keeping it in its native lipid surrounding. Here we present the combined use of SMALPs and LILBID-MS, allowing determination of oligomeric states of membrane proteins of different functionality directly from the native nanodiscs.

## Abstract 2

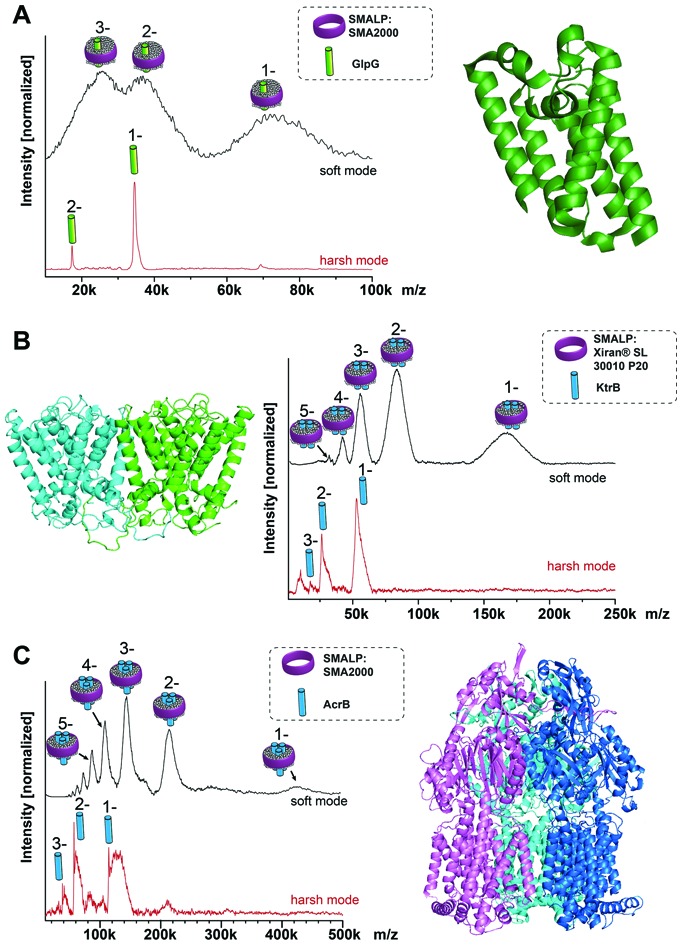
Other than more widely used methods, the use of styrene maleic acid copolymers allows the direct extraction of membrane proteins from the lipid bilayer into SMALPs keeping it in its native lipid surrounding.

## 

Membrane proteins represent a large subclass of the proteome. In comparison to soluble proteins they pose an analytical challenge, as generally an artificial membrane mimetic environment is required to maintain its native state outside of the cellular environment. Popular techniques to achieve this make use of detergent micelles, bicelles, liposomes or nanodiscs, which all aim to keep the hydrophobic membrane protein in solution. One downside of these approaches is that the proteins get heavily delipidated and purified in an artificial surrounding – in the surrogate lipophilic environments used, only certain aspects of the native lipid environment in a cell membrane are mimicked. Depending on the system and the question, this might be sufficient, but in other cases, especially if the annular lipids and/or the native lipid composition are of interest or relevance, another approach is needed. A recent, more sophisticated approach is the use of amphiphilic styrene-maleic acid (SMA)-copolymers,^1^,^2^ which can extract membrane proteins directly from their native cell membrane. These polymer–lipid-combinations – so called styrene maleic acid lipid particles (SMALPs) or native nanodiscs, keep the membrane proteins in their native lipid environment. While this approach has shown to be promising for a lot of analytical methods,^3^–^5^ it poses new challenges for mass spectrometric (MS) analysis of membrane proteins: in contrast to conventional nanodiscs, which utilize a scaffold protein with a defined mass, stoichiometry, and diameter, the SMA polymer exhibits a significant mass distribution and the diameter of the SMALPs and the number of polymers surrounding the protein in the SMALPs are not well defined. In combination, these factors lead to a significant peak broadening of the intact SMALP–protein-complex putting it under the detection threshold if using the established nanoElectroSprayIonization-MS (nESI-MS) protocols for other solubilization techniques. In these protocols generally collisionally induced dissociation (CID) is used to release the protein from its environment, such as attached buffer or detergent molecules. The highly charged nESI ions undergo collisions with inert gas atoms in a collision cell, with CID voltages in native MS in the area of up to 200 V. These voltages are not sufficient to release the intact membrane protein from the stronger binding of the native lipid environment and the SMA copolymer (see Fig. S1, ESI†).

Here we present a study on the applicability of LILBID-MS (Laser Induced Liquid Bead Ion Desorption-MS),^6^ which has already proven successful for the investigation of membrane protein complexes from detergent micelles^7^ and conventional nanodiscs,^8^,^9^ preserving the native oligomeric state for a large number of different proteins.

For LILBID the analyte is transferred into the mass spectrometer in small droplets (50 µm diameter) of the sample solution produced by a piezo-driven droplet generator and is liberated from the aqueous solution by irradiation with a mid-IR laser. This results in lower, more native-like charge states in comparison to nESI. The release of the protein from their attachments stemming from their solubilisation environment is also accomplished by the nanosecond laser pulse. The degree of this laser clean-up can be controlled by the intensity of the laser pulse. In soft mode (∼10 mJ laser pulse power) most attachments remain on the protein, while in harsh mode (typically 23 mJ laser pulse power), the protein is stripped of most of its attachments. This bypasses the need of a collision cell to release the membrane protein and eliminates the risk of collisionally induced unfolding of the protein. A more detailed description of the LILBID technique is given in the ESI.†

To test the general suitability of LILBID-MS for the analysis of membrane proteins in SMALPs, a model system was chosen, consisting of the membrane protein GlpG, a rhomboid protease with known molecular mass of 34.5 kDa, extracted with SMA2000 SMALPs from *Escherichia coli* membranes.^3^ Native nESI-MS and native-PAGE gel results have shown GlpG to be monomeric (Fig. S1, ESI†). The mass spectra in Fig. 1A show that it is possible to transfer the entire GlpG/SMALP construct to the gas phase with the low charge states typical for LILBID. Under soft laser conditions (Fig. 1A top) the entire construct is detected with a distribution of 1 to 3 negative charges. The peak features are quite broad, which is not unexpected and can be attributed to a heterogeneous distribution of polymer and lipids still attached to the protein. The maximum intensity of the broad peak shapes correlates to a mass of 72 kDa. Increasing the LILBID laser power strips the polymer and lipids from the protein, releasing the monomeric GlpG (Fig. 1A bottom). This can be detected at the expected molecular weight of 34.5 kDa, confirming that the protein can be released from the SMALP by laser irradiation. Comparison of both mass spectra shows that the average mass of the broad peak shapes visible at low laser power is about 38 kDa higher than the monomer mass seen at high laser intensities. Previously, a phosphate assay of a GlpG SMALP preparation showed a GlpG : lipid ratio in excess of 1 : 100,^10^ therefore the additional mass is not due to the presence of dimeric GlpG but stems from the native lipids and polymer surrounding the protein. Interestingly, this also shows the ability of LILBID-MS to more accurately estimate the size of the lipid annulus within SMALPs – detecting a GlpG : lipid ratio closer to 1 : 50.

**Fig. 1:**
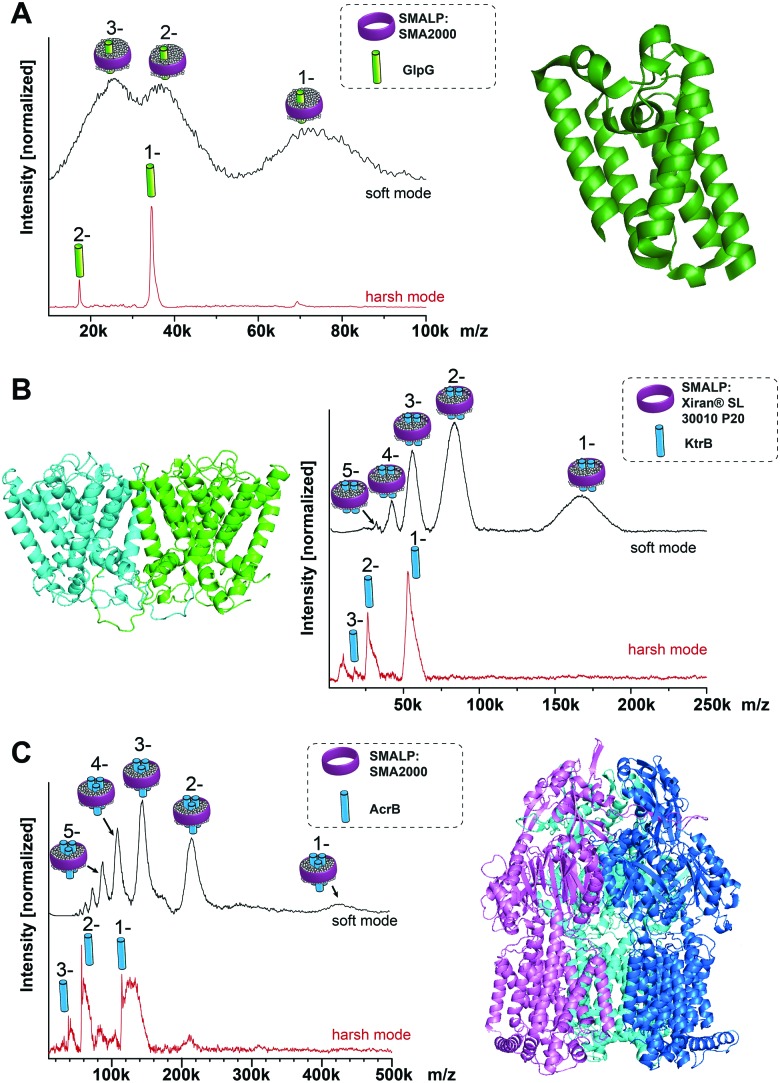
LILBID spectra of membrane proteins of known stoichiometry. (A) Monomeric GlpG; (B) dimeric KtrB; (C) trimeric AcrB. In all cases in soft LILBID mode (low laser power, 10 mJ laser pulse power) and harsh mode (high laser power, 23 mJ laser pulse power) can be compared (top or bottom spectra respectively). Soft laser mode detects the protein/SMALPs-complex, while harsh mode reveals the monomeric protein.

After this promising result, we then explored whether our LILBID/SMALPs approach could be used to investigate membrane proteins with higher oligomeric states. We first tested whether we could detect oligomers of the KtrB protein: the membrane-integral subunit of the high affinity potassium channel KtrAB with a monomeric molecular weight of 54 kDa which is known to form homodimers under native conditions and in detergent micelles.^11^–^13^ Fig. 1B shows, in low laser mode a charge state distribution of 1 to 5 for the KtrB_2_/SMALP complex. This charge distribution again features broad peak shapes with a mean mass of 166 kDa. With increased laser power, the KtrB_2_/SMALP complex is broken apart and the KtrB monomer is released, in some part with a small number of lipids still attached. In contrast to comparable experiments in detergent micelles^11^ the intact KtrB dimer cannot be released from the native SMALPs nanodisc without dissociation. We attribute this to the binding affinities of the polymer entangling the protein and its surrounding lipids being higher than the non-covalent interactions between the KtrB monomers. The additional mass (attributed to contributions from bound lipids and the SMA polymer) for KtrB_2_/SMALP was 62 kDa, clearly a higher mass shift than we observed for the monomeric GlpG.

Next, AcrB, a homotrimeric complex^14^ with a molecular weight of 342 kDa (114 kDa monomer), which is part of the AcrA-AcrB-TolC efflux pump complex, served as even higher oligomer with a bigger molecular mass. In the low laser power mode for the SMALPs sample shown in Fig. 1C, a charge distribution of 1 to 5 is obtained, with the peak maximum of the expected broad mass distribution correlating to a mass of 432 kDa. This corresponds to a mass shift of 90 kDa from the theoretical molecular weight of the trimer. The high laser power mode, again shows only a monomeric AcrB. Interestingly, the amount of attached lipids is noticeably higher than for the previously observed proteins, measured at the same laser power. This could indicate a stronger bond between these lipids and AcrB. Previous LILBID-MS of AcrB in DDM micelles^15^ in comparison allowed the detection of AcrB as a trimer at low to intermediate laser power – likely due to the weaker interactions of the protein with the detergent in contrast to lipid and polymer. Only at higher laser power did the complex dissociate, leading to spectra showing predominantly monomer, similar to the SMALPs measurements at higher laser power. This shows that the energy sufficient to release the AcrB protein complex from the SMALP is higher than for the release from DDM micelles and already dissociates the complex into its monomers. It appears to be a general trend that, in contrast to measurements of membrane protein complexes in detergent micelles, complexes cannot be released intact from the SMALP due to the higher energy input needed to overcome the interactions between the protein complex and its environment.

Postis *et al.* published sedimentation velocity AUC profiles of AcrB SMALPs suggesting a mass of the whole complex exceeding 400 kDa,^4^ which is in good agreement with the mass shifts observed in this work. In the same work they postulated that in the AcrB/SMALPs system, about 80 lipids are needed to cover the entire transmembrane part of the protein complex. The additional weight from these lipids amounts to ∼60 kDa, added to one or several polymer molecules with a mean molecular weight of 7.5 kDa. This correlates well to the mass shifts of 432 kDa that can be observed in the LILBID measurements of this system.

As seen for the oligomeric membrane protein complexes analyzed so far directly from SMALPs, the release of the intact complex with high laser power does not seem possible, differently to the findings using conventional nanodiscs or detergent solubilization. The energy input needed to release the protein within SMALPs is higher than the energy needed to dissociate the protein complex, preventing the direct detection of the free native oligomer. To assess an unknown native oligomeric state of a membrane protein complex it is therefore necessary to find a means to determine how much of the measured mass arises from the polymer/lipid environment. The molecular weight of the protein does not qualify as a good measure to predict the mass shift stemming from polymer and lipids, as it also includes the proteins’ soluble parts which have no influence on the number of lipids attached to the complex. The number of surrounding lipids is likely determined by the space the protein complex takes up in the cell membrane. Hence another approach is necessary. Fig. 2 shows for all the protein/SMALP complexes investigated so far that there is an almost linear correlation between the additional mass observed and the number of known transmembrane helices (TMHs) present. This relationship enables the determination of both the proteins’ oligomeric state and the mass of the lipid/polymer environment.

**Fig. 2:**
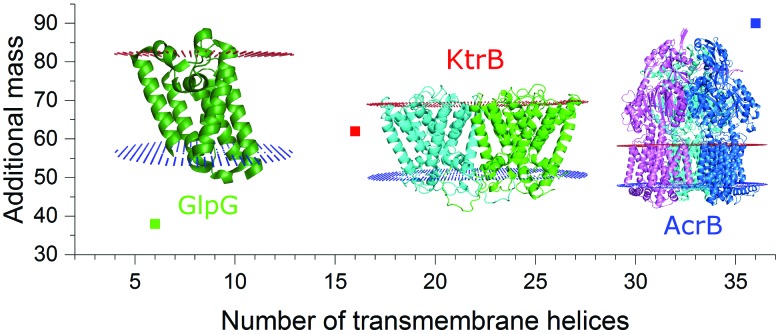
Correlation of the additional SMALP/lipid mass to the number of transmembrane helices present in the respective membrane protein complex.

This brings us into the position to endeavor the investigation of protein complexes of unknown stoichiometries. First, we tested the applicability of the LILBID/SMALPs method on a sodium–solute symporter protein (SSS), a not yet intensively studied protein, suspected to be a proline transporter. The SSS protein (spectra shown in Fig. 3A) has a theoretical molecular mass of 54 kDa and its oligomeric state is unknown. Generally, SSS family members are suggested to either form functional monomers or dimers.^16^–^19^ We further investigated another membrane protein with an unknown oligomeric state: KimA (Fig. 3B), a potassium importer which has a known monomeric molecular weight of 70 kDa.^20^ No native oligomeric state of this protein has been determined up to now.

**Fig. 3:**
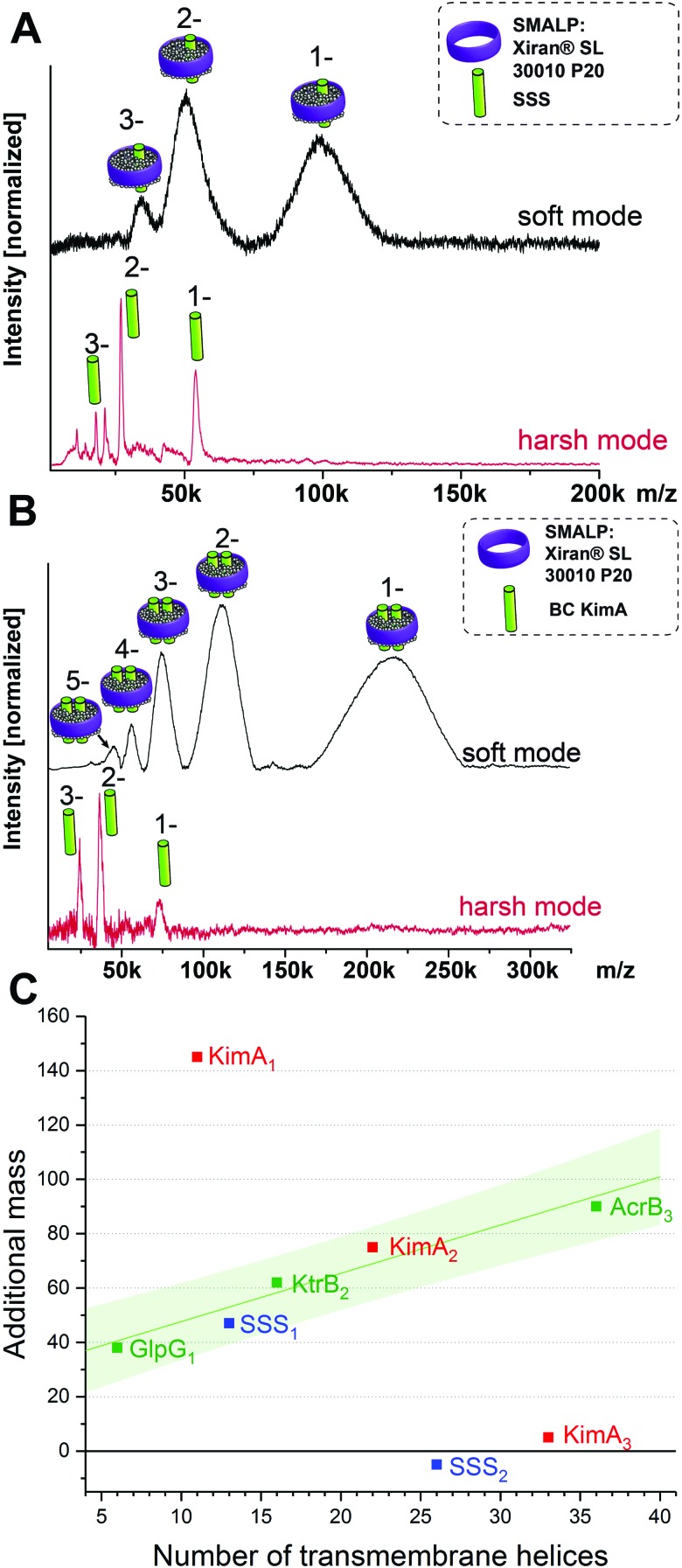
LILBID spectra of SSS (A) and KimA (B) showing the monomer in harsh mode and the protein/SMALPs-complex in soft mode. The oligomeric state of KimA can be determined using a regression of the mass of the surrounding environment in dependence on the number of the transmembrane helices of the protein complex (C). The proteins of known oligomeric state are shown in green, including a linear fit of the data points with the 90% prediction band of the fit shown in light green. The values representing the additional mass for the theoretically possible oligomeric states for SSS (blue) and Kim A (red) are also plotted. For SSS and KimA the monomer and dimer respectively fit well into the 90% prediction band of the fit, while other oligomeric states are significantly outside of the expected range.

As previously, the protein/SMALP complex for the SSS protein is first detected using low laser power. The observed mass spectrum (top in Fig. 3A) shows a charge distribution of 1 to 3 for a mass of 101 kDa. At high laser power the released monomer is present in the spectrum at the expected mass of 54 kDa. This places the average additional mass of the surrounding environment observed at low laser energies at 47 kDa, which is not enough to justify assignment of the SSS protein as dimer. The correlation of the additional mass to the number of transmembrane helices (predicted 13 for the SSS protein monomer) established for the proteins of known oligomeric state also fits the expected range as seen in Fig. 3C (blue data point).

Based on its amino acid sequence a KimA monomer is predicted to contain 11 transmembrane helices. The lower laser power spectrum (top in Fig. 3B) shows a charge distribution of rather broad signals for the protein complex inside the SMALPs, with maximal peak intensities correlating to 218 kDa. The KimA monomer can be detected with higher laser power at the expected molecular weight of 70 kDa. The detected mass of 218 kDa could theoretically be explained by three oligomeric states: a monomer would require a 148 kDa additional mass of lipids and polymers to explain the detected mass, a dimer would require 78 kDa of additional mass and a trimer 8 kDa. All possibilities are plotted into the diagram in Fig. 3C. It becomes immediately clear that only the dimer fits the established correlation, determining KimA as a dimer. Additional control measurements in detergent micelles are in agreement with the oligomeric states determined here (Fig. S3, ESI†).

To conclude, LILBID-MS is the first, and currently the only, mass spectrometric method to determine the oligomeric state of membrane protein complexes in combination with the emerging SMALPs technique. This combination is of high interest, as it allows analysis of membrane proteins in their native lipid environment, encapsulated directly out of their cellular membrane. The release of the intact protein complex from the SMALPs has not been possible, likely due to the strong interactions between the protein and its native lipid environment. Nevertheless, a reliable estimation of the mass of the polymer/lipid environment is possible using the number of transmembrane helices in the protein complex. This allows the unambiguous determination of unknown native oligomeric states. The combination of the two methods is also promising for the analysis of interactions between membrane proteins and their native membrane environment, as we observe that tightly bound lipids can be retained by proteins dissociated from the SMALPs, as seen especially for the case of AcrB. Detecting the number and identity of these tightly bound lipids will be possible with an improvement of the mass resolution of the LILBID instrumentation. More generally, this technique will become an important addition to the emerging membrane protein structural biology investigations using native nanodisc technologies, complementing X-ray crystallography,^5^ cryo-EM,^4^ and hydrogen/deuterium exchange MS^3^ methodologies.

The authors thank David Griwatz for the preparation of the KimA sample in detergent. N. M. is supported by the European Research Council (FP7/2007-2013)/ERC Grant agreement no. 337567. I. H. is funded by the Emmy Noether program (HA6322/3-1). N. M. and I. H. received funding from the Cluster of Excellence Frankfurt (Macromolecular Complexes) and the DFG (German Research Foundation), Collaborative Research Center 807. E. R. is funded by a BBSRC Future Leader Fellowship (BB/N011201/1). A. P. is supported by the Wellcome Trust (109854/Z/15/Z). P. B. received funding from ERC Advanced Grant 294342 and King's College, London.

## Conflicts of interest

There are no conflicts to declare.

## Supplementary Material

Supplementary information
