# Supplementary material for: Native mass spectrometry goes more native: investigation of membrane protein complexes directly from SMALPs
Source: Chem Commun (Camb). 2018 Nov 19;54(97):13702–5. doi: 10.1039/c8cc06284f (PMC6289172; doi:10.1039/c8cc06284f)
Supplement: Supplementary file 1 [file CC-054-C8CC06284F-s001.pdf]

Electronic Supplementary Material (ESI) for ChemComm.
This journal is © The Royal Society of Chemistry 2018

Supporting Information for: Native mass spectrometry goes more native: investigation of membrane protein complexes directly from SMALPs

Nils Hellwig[*a†], Oliver Peetz[a†], Zainab Ahdash[b], Igor Tascón[c], Paula J. Booth[b], Vedrana Mikusevic[c], Marina Diskowski[c], Argyris Politis[b], Yvonne Hellmich[c], Inga Hänelt[c] , Eamonn Reading[b] and Nina Morgner [a]

a. Institute of Physical and Theoretical Chemistry, Goethe University Frankfurt, Max-von-Laue-Straße 7, 60438 Frankfurt, Germany

b. Department of Chemistry, King's College London, 7 Trinity Street, SE1 1DB, London, UK

c. Institute of Biochemistry, Goethe University Frankfurt, Max-von-Laue-Straße 9, 60438 Frankfurt, Germany

† Contributed equally

LILBID-MS. A piezo-driven droplet generator (MD-K-130 from Microdrop Technologies GmbH, Norderstedt, Germany) is used to produce droplets of 50 µm diameter with a frequency of 10 Hz at 100 mbar. Samples are directly loaded into the droplet generator. The generated droplets are transferred to high vacuum and irradiated by an IR laser directly in the ion source. The pulse length is 6 ns with a maximum energy of 23 mJ. The laser is a standard Nd:YAG laser and works at 10 Hz. The wavelength is tuned by a $LiNbO_3$ optical parametric oscillator to 2.94 µm ± 5 nm, the absorbing wavelength of the symmetric and asymmetric O-H stretching vibration of water. The laser power was measured by an optical power meter (PM100D, Thorlabs, Munich, Germany).

The droplet irradiation leads to an explosive expansion of the sample droplet and solvated ions are released and analyzed in a homebuilt time-of-flight setup including a reflectron, operating at 10^{-6} mbar.

The ion source is using ion optics based on a Wiley-McLaren type accelerator. The ions enter the flight tube and are guided towards the detector via a reflectron.

The detector setup is based on a Daly-type detector. Both ion modes can be used. For this work ion detection was done in the negative mode. Spectra processing was done by using the software *Mass*ign[1] based on *LabVIEW*.

LILBID settings. The voltage of the first (repeller) and second plate was set to -4 kV in the ion source. The third plate is grounded. The repeller was pulsed to – 6.6 kV for 370 µs after droplet irradiation. The pulse was applied between 2-50 µs after the droplet irradiation (delayed extraction time). The einzel lenses were set between -2.0 kV up to -3.0 kV. The reflectron was set to -7.2 kV and the TOF operates at 10^{-6} mbar. Post-acceleration was set to +17 kV at the MCP impact surface. Droplet production and laser irradiation was operating at 10 Hz.

GlpG production, purification, and characterization. GlpG was produced and purified into native nanodiscs as previously described[2]. We found that GlpG was pure, folded, thermally stable, and functional within these homogenous native nanodiscs (~14-18 nm in diameter).

Blue native gels were performed as per the manufacturer's instructions using the NativePAGE Novex Bis-Tris gel system (Invitrogen). Native mass spectrometry was performed as previously described[3]. The nESI spectra of GlpG are shown in Supp. Fig. 1+2.

Purification of KtrB-3C-His$_{10}$. *Escherichia coli* strain LB2003[4] was used for the overexpression of *ktrB-C3-his$_{10}$* from *Vibrio alginolyticus* encoded on pBKtrBC3H and generated by FX-cloning.[5] Cells producing *Va*KtrB were grown at 37 °C in 4 L KML medium[6] containing 100 µg/ml ampicillin as a selection marker. Protein production was induced with 0.02 % L-arabinose at an OD_{600} of 1.8. After 1 h cells were harvested and washed with buffer S [420 mM NaCl, 180 mM KCl, and 60 mM Tris-HCl, pH 8] supplemented with 1 mM EDTA, 0.1 mM PMSF and some DNase. Cells were broken with a Laborotory Homogenizer (Stansted Fluid Power) at 1 kbar. The suspension was centrifuged for 15 min at 25,000 × g, and its supernatant was centrifuged overnight at 100,000 × g. The pellet was weighed and suspended to 50 mg/ml in buffer S. Solubilization was reached by the addition of 2.5 % of the SMA polymer Xiran® SL 30010 P20 (Polyscope) over day. Unsolubilized proteins were removed by centrifugation at 200,000 x g for 30 min. The supernatant was incubated with nickel-nitrilotriacetic acid-agarose (Ni^{2+}-NTA) resin over night in the presence of 10 mM imidazole. Subsequently, the Ni^{2+}-NTA was washed with 50 column volumes of buffer W [140 mM NaCl, 60 mM KCl, 20 mM TrisCl, pH 8] containing 50 mM imidazole. *Va*KtrB was eluted with buffer W containing 500 mM imidazole. The protein was loaded onto a Superose Increase 200 10/300 GL column (GE Healthcare) equilibrated to LILBID-MS buffer [20 mM NaCl, 20 mM Tris-HCl, pH 8].

AcrB production and purification (with SMA). A pET15b plasmid containing C-terminally 6x-His tagged wild-type AcrB was transformed into C43(DE3) *Escherichia coli* strain cells. 8 ml of a 200ml overnight LB culture was added to 1L of pre-warmed LB culture containing 100 µg/ml ampicillin and grown at 37 °C until an OD of 0.6-0.8 was reached and then induced with 1 mM IPTG and grown for 2.5 hours at 37 °C.

The cells were then harvested by centrifugation at 4200 *x g* for 30 min and washed with ice cold PBS. Cell pellets were either stored at –80 °C or immediately resuspended in buffer A (50 mM TrisCl, 300 mM sodium chloride, pH 7.4). The resuspension was supplemented with a protease inhibitor tablet (Roche), 100 mM PMSF, 1 µl Benzonase, and 5 mM beta-mercaptoethanol (β-ME). The cell suspension was passed twice through the microfluidizer processor (Microfluidics) at 25,000 psi. Any insoluble material was removed by centrifugation at 20,000 x g for 30 min at 4 °C. Membranes were then pelleted by centrifugation at 200,000 *x g* for 1 hr at 4 °C. Membrane pellets were then resuspended to 40 mg ml^{-1} in ice-cold buffer B (50 mM TrisCl, 300 mM sodium chloride, 10% glycerol, pH 7.4). The resuspension was supplemented with a protease inhibitor tablet (Roche) and 100 mM PMSF, and then homogenized using a Potter-Elvehjem Teflon pestle and a glass tube.

AcrB was solubilized and purified from membranes with SMA 2000 to form AcrB native nanodiscs. To solubilize the membranes, SMA 2000 co-polymer powder was added to the suspension at a final concentration of 2.5 % (w/v) and incubated with gentle agitation at room temperature for 2 hours. Insoluble material was pelleted by centrifugation at 100,000 x g for 30 min at 4 °C.

1 ml super nickel NTA agarose affinity resin (Generon), equilibrated in buffer B supplemented with 20 mM Imidazole, was added to the solubilized membrane suspension and left to equilibrate overnight at 4 °C with gentle agitation. The beads were then transferred to a gravity-flow column and washed with 10 column volumes (CVs) of buffer B supplemented with 20 mM Imidazole and a further 10 CVs of buffer B supplemented with 50 mM Imidazole. The protein as then eluted with 5 CVs of buffer B supplemented with 500 mM imidazole. The protein samples were then buffer exchanged into buffer B using a PD-10 desalting column (GE Healthcare), flash frozen in liquid nitrogen, and stored at –80 °C.

To determine the homogeneity of the protein sample, an aliquot was injected into a Superdex 200 10/300 GL size exclusion chromatography (SEC) column (GE Healthcare) equilibrated in buffer B. AcrB purification was assessed using SDS-PAGE electrophoresis and a Markwell-Lowry assay was used to determine the protein concentrations.

Purification of SSS-3C-His$_{10}$. *Escherichia coli* strain C43(DE3) was used for the overexpression of *SSS-C3-his$_{10}$* from *Hyphomonas neptunium* encoded on pBEctIC3H and generated by FX-cloning.[7] Cells producing EctI were grown at 37 °C in 6 L LB medium containing 100 µg/ml ampicillin as a selection marker. Protein production was induced with 0.002% L-arabinose at an OD_{600} of 1.3. After 1 h cells were harvested and washed with buffer S [600 mM NaCl, and 50 mM Tris-HCl, pH 8] supplemented with 1 mM EDTA, 0.1 mM PMSF, 0.3 mM Benzamidine and some DNase. Cells were broken with a Laborotory Homogenizer (Stansted Fluid Power) at 1 kbar. The suspension was centrifuged for 15 min at 35,000 × g, and its supernatant was centrifuged overnight at 100,000 × g. The membrane pellet was weighed and suspended to 50 mg/ml in buffer W [140 mM NaCl, 60 mM KCl and 20 mM Tris-HCl, pH 8]. Solubilization was reached by the addition of 2.5% of the SMA polymer Xiran® SL 30010 P20 (Polyscope) over day. Unsolubilized proteins were removed by centrifugation at 200,000 *x* g for 30 min. The supernatant was incubated with nickel-nitrilotriacetic acid-agarose (Ni^{2+}-NTA) resin over night in the presence of 10 mM imidazole. Subsequently, the Ni^{2+}-NTA was washed with 50 column volumes of buffer W [140 mM NaCl, 60 mM KCl, 20 mM TrisCl, pH 8] containing 50 mM imidazole. SSS was eluted with buffer W containing 500 mM imidazole. The protein was loaded onto a Superose6 Increase 200 10/300 GL column (GE Healthcare) equilibrated to LILBID-MS buffer [20 mM NaCl, 20 mM Tris-HCl, pH 8].

Purification of KimA. *Escherichia coli* C43(DE3) cells were grown for 1.5 h after induction with 0.002 % arabionse at 37 °C to produce KimA. After harvesting, the cell pellet was resuspended in 50 mM Tris pH 8, 100 mM KCl, 400 mM NaCl, 1 mM EDTA supplemented with 0.5 mM PMSF, 1 mM benzamidine and DNase I, and the suspension was passed through a homogenizer at a pressure of 1kbar to disrupt the cells. The cell extract was centrifuged at 15,000 x g for 15 minutes to remove unbroken cells and debris. Membranes were collected after centrifugation at 180,000 x g for 3h. The membrane pellet was solubilised with 2 % SMA co-polymer Xiran SL30010 P20 (2:1 molar ratio of styrene:maleic acid), provided by Polyscope Polymers B. V., in 50 mM Tris pH 8, 100 mM KCl, 400 mM NaCl, at 4 °C overnight. After overnight incubation the solution was centrifuged at 180,000 x g for 30 minutes to remove unsolubilised particles. The supernatant was incubated with Ni-NTA overnight. Then the resin was washed out with 50 column volumes with solubilisation buffer supplemented with 50 mM imidazole to remove any unbound sample. The protein was eluted using 500 mM imidazole in the solublisation buffer, and further purified by size exclusion chromatography using a Superose 6 10/300 GL column (GE Healthcare) previously equilibrated with 50 mM Tris pH 8, 100 mM KCl. Fractions containing the protein were pooled and the sample was desalted prior to LILBID analysis.

Preparation of SMA polymer Xiran® SL 25010 P20 (Polyscope). SMA Copolymer was diluted to a conc. of 6 mg/mL *(v/v)* in unadjusted 50 mM Tris. The resulting pH of the stock solution was 8.4.[8]

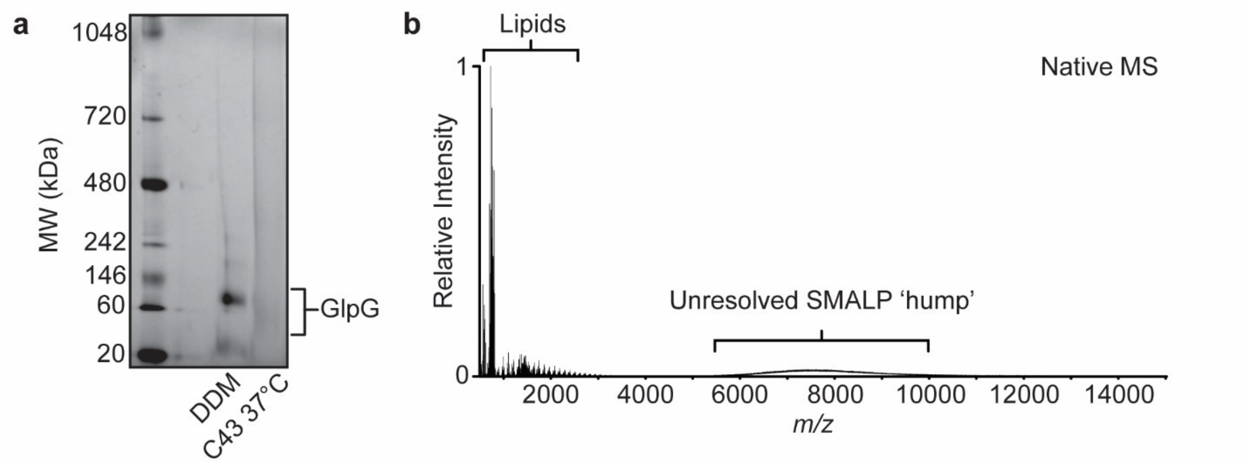

Supp. Figure 1. (**a**) Native PAGE gel analysis of GlpG purified in DDM detergent micelles and in native nanodiscs reveal GlpG travels at a soluble calibrated mass of approximately 70 kDa – the native nanodisc samples cannot be resolved as a single band but run as a smeared band as shown previously for nanodisc membrane protein samples.[8] Membranes proteins have been shown to require significant correction (they run 1.8x higher than their native mass) on blue Native PAGE gels due to their enhanced Comassie binding compared to soluble proteins.[9] Once corrected, Native PAGE reveals GlpG to likely be monomeric in the native nanodiscs as it is in DDM detergent micelles. (**b**) Native mass spectrometry was performed at the highest collisionally induced dissociation (CID) energies possible on a commercially available Synapt G2Si High Definition mass spectrometer (Waters), revealing dissociated lipids from the GlpG native nanodiscs (SMALPs) and an unresolvable region between 5500-10000 *m/z* proposed to be intact GlpG native nanodiscs.

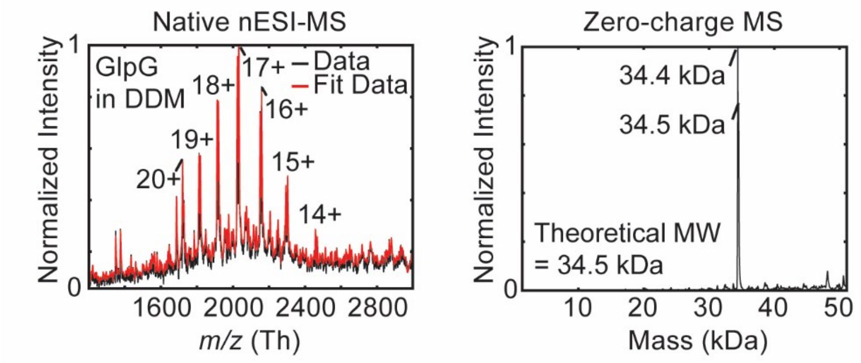

Supp. Figure 2. Native nano-electrospray (nESI) mass spectrometry of GlpG purified in DDM detergent micelles reveals GlpG to be monomeric. Mass spectra was acquired at a collisionally induced dissociation (CID) energy of 180 V, to remove bound DDM detergent in the gas phase, on a commercially available Synapt G2Si High Definition mass spectrometer (Waters). Data was analyzed using UniDec software[10].

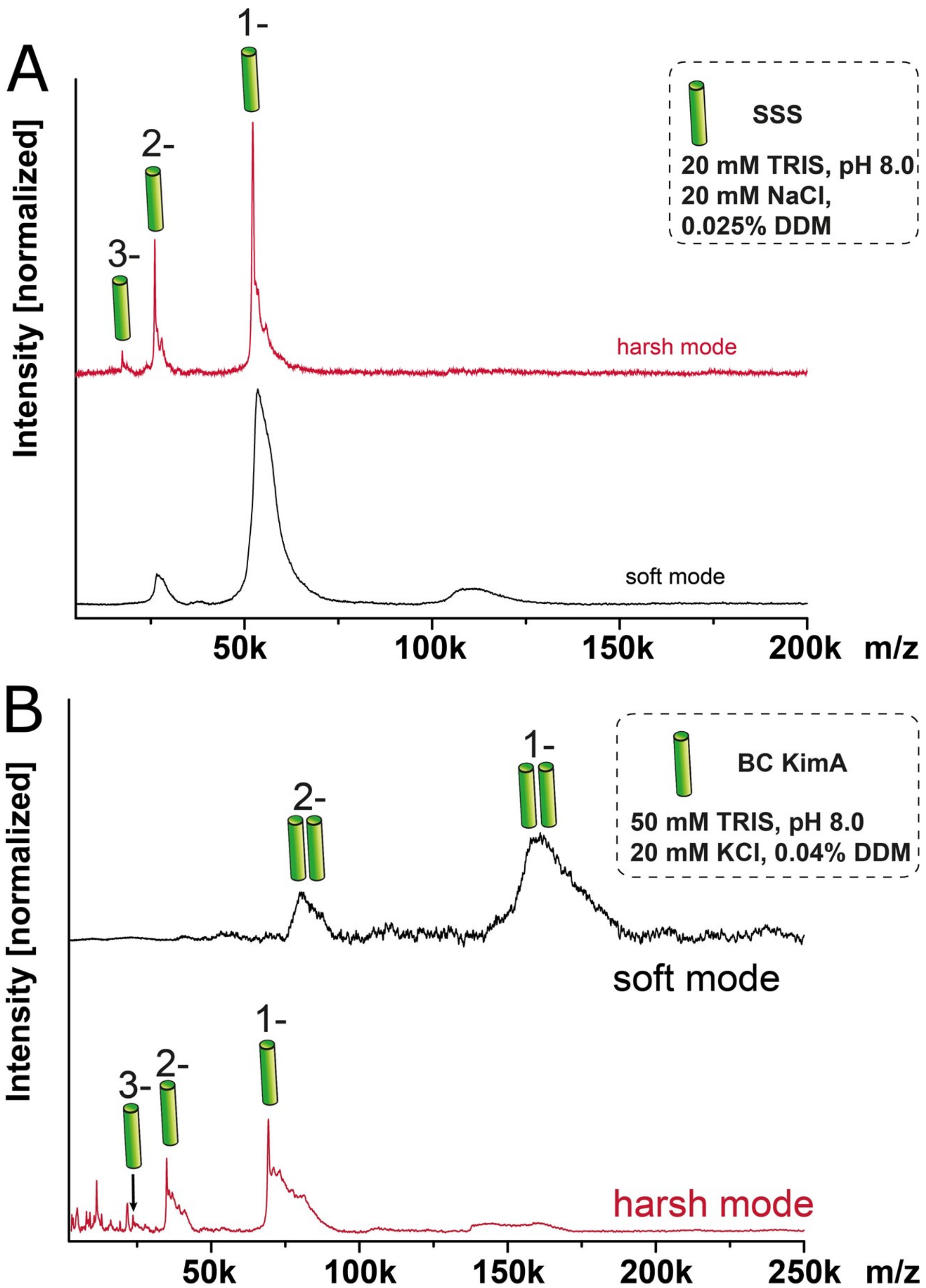

Supp. Figure 3. LILBID-MS of KimA solubilized in DDM micelles. In agreement to the SMALPs spectra, for SSS protein (**A**), the monomeric state can be confirmed from the detergent spectra. The KimA dimer can be detected in soft laser mode, while in harsh mode the complex is dissociated into monomers (**B**).

Literature

1. N. Morgner and C. V. Robinson, *Anal Chem*, 2012, **84**, 2939-2948.
2. E. Reading, Z. Hall, C. Martens, T. Haghighi, H. Findlay, Z. Ahdash, A. Politis and P. J. Booth, *Angew Chem Int Ed Engl*, 2017, **56**, 15654-15657.
3. A. Laganowsky, E. Reading, J. T. Hopper and C. V. Robinson, *Nat Protoc*, 2013, **8**, 639-651.
4. S. Stumpe and E. P. Bakker, *Arch Microbiol*, 1997, **167**, 126-136.
5. E. R. Geertsma, *Methods Mol Biol*, 2013, **978**, 133-148.
6. N. Kröning, M. Willenborg, N. Tholema, I. Hänelt, R. Schmid and E. P. Bakker, *J Biol Chem*, 2007, **282**, 14018-14027.
7. E. R. Geertsma, *Methods Mol Biol*, 2014, **1116**, 153-164.
8. M. Alami, K. Dalal, B. Lelj-Garolla, S. G. Sligar and F. Duong, *EMBO J*, 2007, **26**, 1995-2004.
9. P. G. Crichton, M. Harding, J. J. Ruprecht, Y. Lee and E. R. Kunji, *J Biol Chem*, 2013, **288**, 22163-22173.
10. D. J. Reid, J. M. Diesing, M. A. Miller, S. M. Perry, J. A. Wales, W. R. Montfort and M. T. Marty, *J Am Soc Mass Spectrom*, 2018.
